# Pathogenesis of Chronic Cardiorenal Syndrome: Is There a Role for Oxidative Stress?

**DOI:** 10.3390/ijms141123011

**Published:** 2013-11-20

**Authors:** Speranza Rubattu, Silvia Mennuni, Marco Testa, Mara Mennuni, Giorgia Pierelli, Beniamino Pagliaro, Erica Gabriele, Roberta Coluccia, Camillo Autore, Massimo Volpe

**Affiliations:** 1 Department of Clinical and Molecular Medicine, School of Medicine and Psychology, Sapienza University of Rome, Ospedale S. Andrea, Rome 00189, Italy; E-Mails: silvia.mennuni@gmail.com (S.M.); mar.testa@tin.it (M.T.); giorgia.pierelli@alice.it (G.P.); benpag@libero.it (B.P.); erica.gabriele85@gmail.com (E.G.); roberta.coluccia@libero.it (R.C.); camillo.autore@uniroma1.it (C.A.); massimo.volpe@uniroma1.it (M.V.); 2 IRCCS Neuromed, Pozzilli (Isernia) 86077, Italy; 3 Department of Biochemical Sciences A. Rossi Fanelli, Sapienza University of Rome, Rome 00185, Italy; E-Mail: mara.mennuni@gmail.com

**Keywords:** heart failure, renal failure, oxidative stress, mitochondrial dysfunction, renin-angiotensin-aldosterone system, sympathetic nervous system, inflammation

## Abstract

Cardiorenal syndrome is a frequently encountered clinical condition when the dysfunction of either the heart or kidneys amplifies the failure progression of the other organ. Complex biochemical, hormonal and hemodynamic mechanisms underlie the development of cardiorenal syndrome. Both *in vitro* and experimental studies have identified several dysregulated pathways in heart failure and in chronic kidney disease that lead to increased oxidative stress. A decrease in mitochondrial oxidative metabolism has been reported in cardiomyocytes during heart failure. This is balanced by a compensatory increase in glucose uptake and glycolysis with consequent decrease in myocardial ATP content. In the kidneys, both NADPH oxidase and mitochondrial metabolism are important sources of TGF-β1-induced cellular ROS. NOX-dependent oxidative activation of transcription factors such as NF-kB and c-jun leads to increased expression of renal target genes (*phospholipaseA2, MCP-1* and *CSF-1, COX-2*), thus contributing to renal interstitial fibrosis and inflammation. In the present article, we postulate that, besides contributing to both cardiac and renal dysfunction, increased oxidative stress may also play a crucial role in cardiorenal syndrome development and progression. In particular, an imbalance between the renin-angiotensin-aldosterone system, the sympathetic nervous system, and inflammation may favour cardiorenal syndrome through an excessive oxidative stress production. This article also discusses novel therapeutic strategies for their potential use in the treatment of patients affected by cardiorenal syndrome.

## 1. Introduction

Maintenance of blood volume, vascular tone, and hemodynamic stability depends on a set of well-balanced interactions between the heart and kidneys. Both organs show endocrine functions with interdependent physiological actions, mostly related to natriuretic peptides and the renin-angiotensin-aldosterone system (RAAS). In addition, the sympathetic nervous system (SNS) plays a role in modulating the functional relationship between the two organs. Therefore, it is not surprising that dysfunction of either organ can severely compromise the function of the other.

The cardiorenal syndrome (CRS) is defined as a condition in which either cardiac or renal dysfunction amplifies the failure progression of the other organ, ultimately leading to increased cardiovascular morbidity and mortality.

The syndrome has been classified into five subtypes based on the organ primarily involved (heart or kidney) and on whether the failure is acute, chronic or secondary [1–3].

While hemodynamic derangements (elevated venous pressure, elevated intra-abdominal pressure, low cardiac output, hypotension) could explain the adverse relationship between heart and kidneys during an acute failure of either one, the interpretation of the complex physiological, biochemical, and hormonal derangements (RAAS, natriuretic peptides) encompassing the chronic CRS remains poorly understood.

The classical Guytonian model [4], which describes heart/kidney interaction by means of cardiac output, regulation of extracellular fluid volume, blood pressure, and renal sodium handling, appears unable to explain the profound organs derangements, the remodeling and the progression of the dysfunction observed in both organs in chronic CRS. Furthermore, chronic CRS, once established, leads to accelerated atherosclerosis [5,6], cardiac hypertrophy [7], microangiopathy [8], increased arterial stiffness [9] and coronary calcifications [10].

The present review article discusses evidence supporting the potential contribution of oxidative stress to CRS development and progression, mainly based on the key role of this mechanism in both heart and renal failure conditions when considered separately.

## 2. Oxidative Stress and Its Impact on Cellular Damage

Oxidative stress is defined as a result of an imbalance between oxidants and antioxidants in favour of the former that potentially leads to cell injury [11]. Oxidative stress occurs when the formation of reactive oxygen species (ROS) exceeds the body’s ability to metabolize them, or when the antioxidant defense mechanisms are depleted. ROS are oxygen-derived small molecules, comprising oxygen radicals’ superoxide, hydroxyl, peroxyl, alkoxyl and non-radicals, such as hydrogen peroxide (H_2_O_2_). ROS generation occurs as by-product in several cellular processes. The mitochondrial respiratory chain activity is responsible for most of the ROS production in aerobiosis.

The multi-subunit transmembrane nicotinamide adenine dinucleotide phosphate (NADPH) oxidase proteins (NOXs) also play a relevant role in ROS production. They utilize NADPH as an electron donor to reduce oxygen and produce low levels of superoxide anion (O_2_^−^) and H_2_O_2_. Out of the seven oxidase family members (NOX 1-5 and dual oxidase 1-2), NOX1, NOX2, NOX4, and NOX5 are expressed in the cardiovascular system. NOX2 and NOX4 are the major isoforms present in cardiomyocytes. NOX2 activation requires the recruitment of several cytosolic subunits (p47phox, p67phox, p40phox and Rac1) which bind to flavocytochrome mainly to induce superoxide anion (O_2_^−^) production. NOX2, found predominantly in the sarcolemma and T-tubules, is activated by G-protein coupled receptors. NOX4 activation, primarily regulated by levels of its expression, mainly produces H_2_O_2_. It is localized in the endoplasmic reticulum and perinuclear regions of cardiomyocytes, although a mitochondrial location has also been suggested [12–14].

High levels of oxygen radicals inactivate mitochondrial enzymes, cause DNA damage and, by interacting with both DNA repair enzymes and transcription factors, lead to cell death.

Inactivation of the endothelium-derived relaxing factor nitric oxide (NO) is an important secondary ROS effect. Superoxide anion O_2_^−^ reacts with NO and inactivates its beneficial effect by forming a very powerful oxidant and nitrosating agent, the peroxynitrite (ONOO^−^). The latter contributes to oxidative stress by oxidizing lipids, DNA, and proteins [15].

In addition, ROS production can lead to “ROS-induced ROS release”, a vicious circle in which ROS species activate the permeability of mitochondrial pores leading to mitochondrial dysfunction and to further ROS release [16].

Interestingly, ROS can damage mitochondrial macromolecules either at or near the site of their formation. Among them, the mitochondrial DNA (mtDNA) could be a major target for ROS-mediated damage for several reasons. First, mitochondria do not have the chromatin organization complex consisting in histone proteins which represent a protective barrier against ROS. Secondly, mtDNA has limited repair ability against DNA damage. Finally, since a large part of the superoxide anion O_2_^−^ produced in mitochondria cannot pass through the membranes, ROS damage is largely contained within the mitochondria [17].

These mechanisms are associated with reduced mtDNA copy number and with a parallel decrease of mtDNA-encoded gene transcripts, which have been associated with reduced activity of mitochondrial complexes I, III, and IV (all containing subunits encoded by mtDNA). In contrast, complex II activity remains unchanged [18].

Oxidative damage may also affect critical steps of Krebs cycle and mtDNA polymerase γ, slowing mtDNA replication and eventually leading to inhibition of oxidative phosphorylation [19].

## 3. Role of Oxidative Stress in Heart Failure (HF)

Normal cardiac function requires high and continuous ATP supply. Being that mitochondria are the major source of ATP production, it is apparent that mitochondrial and cardiac functions are closely related to each other [20].

Strong evidence from both *in vitro* and animal studies shows that several pathways are dysregulated in HF, leading to increased oxidative stress production and to cardiac damage.

First of all, a metabolic shift from fatty acid (FA) oxidation to glycolysis has been reported in cardiomyocytes during HF. In a normal heart, most of the ATP is produced by FA oxidation whereas the remaining part is provided by oxidizing pyruvate, as an end product of glycolysis or derived from lactate [21]. Both pyruvate and FA oxidation pathways are located within the mitochondrial matrix. During HF progression myocardial ATP content decreases, dropping to 60%–70% of normal levels [22–25]. This drop is due to a decrease in mitochondrial oxidative metabolism and it is balanced by a compensatory increase in glucose uptake and glycolysis [25,26].

The shift in the energy source within the cells may result in altered ATP yield, since glycolysis produces less ATP per substrate mole as compared to FA oxidation. Although the glycolitic rate is increased, it is insufficient to supply the energy demands of the failing heart.

The reduced oxidative metabolism leads to accumulation of free FA in cardiomyocytes, creating a self-perpetuating mechanism of ever-increasing oxidative stress and causing deleterious effects within the heart. Either lipotoxicity of circulating FA or the intracellular lipid accumulation contributes to mitochondrial oxidative stress through the activation of protein kinase C, and causes endoplasmic reticulum stress [27]. The progressive decrease of ATP production is linked to both decrease of FA oxidation and reduction of mitochondrial respiration, due to electron transport chain (ETC) defects [28].

Several alterations in ETC components have been described in different stages of HF [29,30].

In particular, decreased activities of complexes III and IV [31], alterations in the components of the phosphorylation apparatus, decreases in the amount and activity of ATP synthase [32], were reported during HF. The altered mitochondrial ETC is a known source of ROS. The decrease in functional respirasomes in HF causes a further drop in oxidative phosphorylation, associated to an increased electron leakage and superoxide generation in complexes I and III. ROS production causes a vicious circle by amplifying the ETC dysfunctions [33].

**Figure 1:**
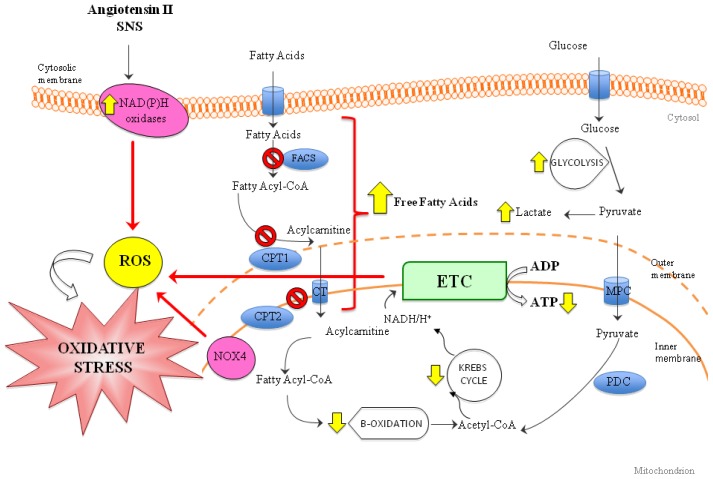
Mitochondrial centrality in cardiomyocyte metabolism. During heart failure (HF), a shift in energy metabolism has been observed in both *in vitro* and animal studies. β-oxidation is reduced, and free fatty acids are not transported into the mythocondrion and accumulate into the cytosol, thus activating the stress response. Impairment of fatty acid oxidation may also be secondary to changes in the activity and kinetic properties of CPT, which is involved in the controlling step of long-chain fatty acids entry into the mitochondria. Both lipotoxicity of circulating fatty acids and intracellular accumulation of lipids contribute to mitochondrial oxidative stress increase. The rise in glycolysis causes an increase of lactate production. The change in energy source leads to a fall in the amount of ATP production. Moreover, ETC dysfunction, the main source of ROS, is one of the major events that leads to increased oxidative stress in HF. RAAS and SNS pathways, by activating NADPH oxidase, also contribute to create a condition of increased oxidative stress within the cell. Legend: FACS = Fatty acyl-coA synthase. CPT1/CPT2 = Carnitine palmitoyltransferase 1/2. MPC = mitochondrial pyruvate carrier. PDC = Pyruvate dehydrogenase. ETC = Electron transport chain. NOX4 = NADPH oxidase 4. ROS = Reactive oxygen species. RAAS = renin-angiotensin-aldosterone system. SNS = sympathetic nervous system.

Apart from the above described changes in the energy metabolism, RAAS and SNS activation also contributes to maintain and amplify the oxidative stress in HF. Angiotensin II activates NADPH oxidase as the primary source of ROS, causing mitochondrial dysfunction [34]. Both NOX4 and NOX2 are upregulated by angiotensin II in a mitochondrial ROS-independent and -dependent manner, respectively [35], suggesting a close relationship between the two sources (Figure 1).

HF is accompanied by adaptive reactions, including the increase of orthosympathetic tone. In this regard, Rosca *et al.* proposed an elegant molecular model in which the increase in adrenergic drive caused a decrease in functional respirasomes and led to mitochondrial dysfunction and progressive decrease in cardiac performance [28].

Once produced, ROS become responsible for several negative effects in the failing heart. They are involved in cardiac remodelling, cardiomyocyte contractility, ion transport and calcium handling. In addition to their detrimental damaging effects, mitochondrial ROS play an important role in intracellular signalling by triggering multiple cellular pathways and the transcriptional activation of selected nuclear genes, finally eliciting transcriptional reprogramming [12,36].

The oxidative alterations causing the decreased activity of ETC complexes reported in severe HF potentially enhance the severity of the energy deficit with a further oxidative stress increase, finally leading to degradation of the oxidized complexes. Oxidative damage of myofibrillar proteins decreases calcium sensitivity, thus interfering with muscle contractile performance [37].

ROS have also been shown to activate matrix metalloproteinase (MMP) in cardiac fibroblasts [38]. Myocardial MMP activity is increased in the failing heart [39]. Prolonged MMP activation might influence the structural properties of the myocardium by providing an abnormal extracellular environment for myocytes. Importantly, it has been demonstrated that the **·**OH scavenger dimethylthiourea inhibits the activation of MMP2 along with the development of left ventricular remodelling and failure [17].

Furthermore, the release of several mitochondria-specific proteins from the intermembrane space, including cytochrome c, endonuclease G (EndoG), apoptosis-inducing factor (AIF) and second mitochondria-derived activator of caspase (Smac), is crucial in the early triggering events of the apoptotic pathway leading to caspase activation, nuclear DNA fragmentation, and cell death [40]. The release of EndoG and AIF, and their translocation to the nucleus promote nuclear DNA degradation, even in the absence of caspase activation [41]. As previously noted, higher ROS concentrations activate stress kinases like c-Jun *N*-terminal kinase (JNK) and p38-mitogen activated protein kinase (MAPK) [42]. JNK activation may link the hypertrophy to the mitochondrial dysfunction observed in HF. In fact, JNK activation not only promotes cardiomyocyte hypertrophy but also activates autophagy, through Bcl-2 and 19-KDa interacting protein-3 (BNIP3), which ultimately leads to apoptosis and mitochondrial selective autophagy (mitophagy) [43,44].

In this regard, Vacek *et al*. showed that an increased level of mitophagy may in turn lead to MMP activation [45].

## 4. Role of Oxidative Stress in Kidney Damage and Failure

Within the kidneys, ROS generation increases in response to specific stimuli, including Angiotensin II [46–49] and aldosterone [50,51], and it influences a number of physiologic processes.

Angiotensin II appears to act preferentially in tubular epitelial cells, whereas recent studies suggested a role of aldosterone in podocyte injury [52].

NOX enzymes are the primary source of ROS in vascular smooth cells in both kidney cortex and medulla [53,54]. Upon stimulation by angiotensin II and aldosterone, cytosolic subunits of NAD(P)H oxidase can translocate into the mitochondrial membrane and increase ROS production.

At least three different NOX isoforms are expressed in the kidney cortex: NOX4, NOX2 and NOX1 [55–58]. Although no strict comparisons have been performed, NOX4 appears to be the most abundantly expressed renal isoform. NOX4 is predominantly localized in renal tubular cell [55,56], but it can also be found at lower levels in other cell types, including glomerular mesangial cells [51,59].

The proposed function of NOX-derived ROS in the kidneys can be classified into three major categories: regulation of renal blood flow, alteration of cell fate and regulation of gene expression. The key mechanism by which ROS regulate renal blood flow is the reaction of superoxide anion O_2_^−^ with NO, which limits its relaxing effect in afferent arterioles [49,60].

Under physiological conditions, NO maintains endothelial function, causes vasodilatation of the afferent arteriole, thus increasing renal blood flow, blunts tubuloglomerular feedback, promotes pressure natriuresis and scavenges low ROS concentrations [61]. As mentioned before with regard to the heart, under conditions of increased oxidative stress superoxide anion O_2_^−^ reacts with NO to form ONOO^−^. The accumulation of ONOO^−^ can lead to cascade reactions that result in vasoconstriction, inflammation and impaired vascular and renal functions.

Under conditions of oxidative stress, decreased NO inhibits cytochrome P450 enzymes and favours the production of vasoconstrictor molecules. High concentrations of NO normally inhibit cyclooxygenases, whereas the decrease of NO enhances their activity [62]. Cyclooxygenases are involved in the production of the vasoconstrictor thromboxane A2 (TxA2), as well as in the synthesis of vasodilators such as prostacyclin-2 and prostaglandin E2. Thus, O_2_^−^ and ONOO^−^ enhance thromboxane synthase activity and TxA2 production, while inhibiting prostacyclin synthase and prostacyclin-2 production, ultimately leading to an imbalance between vasoconstrictors and vasodilators [63].

NOX-derived ROS can alter renal cell fate by enhancing epithelial-mesenchymal transition (EMT) [64,65], presumably through MAP kinase activation, by inducing mesangial cell apoptosis [48] and by promoting cellular hypertrophy through activation of ERK1/ERK2 pathway [46,66].

Tissue fibrosis is a common process involved in the response to chronic stress and injury. It represents the result of several phenomena including EMT, activation of fibroblasts to produce extracellular matrix, recruitment of inflammatory cells, and cellular regeneration at sites of damage. ROS activate a pro-inflammatory and pro-fibrotic state via both cytokines and the transforming growth factor β (TGF-β). The latter has been demonstrated to induce EMT and is thought to be one of the major causes of renal fibrosis [67–69].

EMT of tubular epithelial cells is characterized by loss of epithelial properties and by gain of excessive deposition of extracellular matrix-producing myofibroblast characteristics [70,71].

Yang and Liu demonstrated that EMT is an orchestrated and highly regulated process involving four key steps: (1) loss of epithelial cell adhesion; (2) *de novo* α-smooth muscle actin (α-SMA) expression and reorganization; (3) disruption of tubular basement membrane; (4) enhanced cell migration and invasion into the interstitium [72]. It was also demonstrated that TGF-β1 signalling is sufficient to induce EMT in cultured epithelial cells [73].

In conclusion, ROS may play a role in TGF-β1–induced EMT in renal tubular epithelial cells through the activation of either Smad or MAPK pathways. The inhibition of NADPH oxidase and mitochondrial electron transfer chain subunit I, as well as of antioxidants, significantly reduce TGF-β1-induced ROS generation, suggesting that both NADPH oxidase and mitochondrial metabolism are important sources of TGF-β1-induced cellular ROS [64].

**Figure 2:**
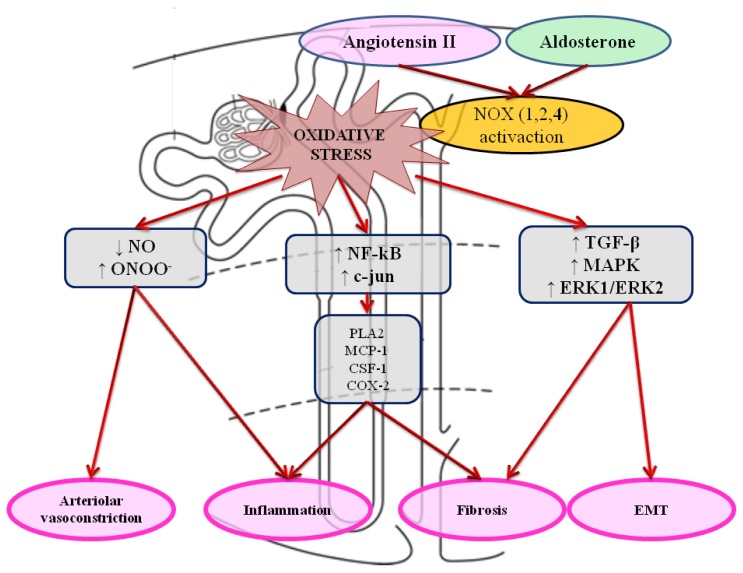
Molecular mechanisms involved in kidney damage and failure. Angiotensin II and Aldosterone represent the main stimuli of NOX enzymes activation, the primary source of ROS in the kidney. Oxidative stress acts through three major molecular pathways: (1) reduction of NO availability and increased ONOO^−^ formation; (2) increased inflammation by NF-κB and c-jun activation; (3) TGF-β, MAPK and ERK1/ERK2 activation. The final effects are: arteriolar vasoconstriction, inflammation, fibrosis and EMT, all underlying progressive kidney damage. Legend: NOX = NADPH oxidase, ROS = Reactive oxigen species, NO: nitric oxide; ONOO^−^: peroxynitrite, NF-kB: nuclear factor kappa-light-chain-enhancer of activated B cells; PLA2: Phospholipases A2; MCP-1: monocyte chemotactic protein-1; CSF-1: colony-stimulating factor-1; COX-2: cyclooxygenase-2; TGF-β: transforming growth factor*-*beta, MAPK: *mitogen-activated protein kinases*; ERK1/ERK2: extracellular-signal-regulated kinases 1/2; EMT: epithelial-mesenchymal transition.

NOX-dependent oxidative activation of transcription factors such as NF-kB [74] and c-jun [75] leads to increased expression of renal target genes (*phospholipaseA2, MCP-1* and *CSF-1, COX-2*) [74,76,77]. Expression of these genes promotes renal interstitial fibrosis and inflammation (Figure 2).

Interestingly, recent evidence has shown that uncoupling proteins (UCPs) play an important role in the regulation of ROS production and inflammation [78]. UCPs are encoded by nuclear DNA and are located at the level of the mitochondrial inner membrane. Their primary function is thought to be the translocation of protons from the intermembrane space to the mitochondrial matrix [79]. Selected *UCP2* gene variants (such as the -*866G* to A gene promoter transition) are significantly associated with chronic kidney disease (CKD) and they may be informative for prediction of genetic risk for CKD [80]. UCPs are also attracting interest as potential therapeutic targets in a number of important diseases [81].

It is worth noting that the clinical implications of the above-described molecular mechanisms, responsible for increased oxidative stress and inflammation within the kidneys, have been clearly shown in the context of human CKD, with particular regard to hemodialytic patients [82–84]. In fact, previous studies have shown that oxidative stress and inflammation are progressively enhanced with advancing stages of CKD [85–87].

## 5. Implications of Increased Oxidative Stress in Chronic Cardiorenal Syndrome

Renal dysfunction in HF has been attributed to biochemical, hormonal, and hemodynamic factors, coupled with pharmacological interventions [81,88] resulting in renal sodium and water retention, which lead to extracellular fluid expansion [89].

Several molecules modulate cardiovascular hemodynamic and their dysregulation can induce cardiorenal damage. Although these adaptive mechanisms are adequate to maintain cardiac output at normal levels during the initial development of CRS, clinical data suggest that they ultimately become maladaptive and result in a progressive decrease in both cardiac and renal performances.

RAAS, SNS and inflammation are the most important mechanisms that, when dysregulated, may lead to cardiorenal damage. Hence, activation of these mechanisms leads to a vicious cycle in which all of them synergize and activate each other, resulting in worsening of both cardiac and renal functions [90–92].

One of the most deleterious effects of RAAS stimulation is NADPH-oxidase activation, resulting in increased ROS formation [93]. This has been documented in endothelial cells, vascular smooth muscle cells [94], renal tubular cells [95], cardiomyocytes [96,97]. Angiotensin-converting enzyme (ACE) inhibition mitigates ROS formation [98] and increases nitric oxide (NO) bioavailability in patients with coronary artery disease [99]. Moreover, Angiotensin II is involved in the vascular inflammatory responses via the NF-κB pathway [100,101]. Finally, RAAS interacts with the SNS through complex mechanisms that can be controlled by ACE-inhibition [102].

Excessive sympathetic activity can induce cardiomyocyte apoptosis, hypertrophy, and focal myocardial necrosis [103]. SNS also contributes to RAAS activation by directly stimulating renin release. Interestingly, noradrenaline induces hypertrophy of cultured cardiomyocytes through superoxide anion O_2_^−^ production [104]. Prolonged SNS over-activity has a growth-promoting effect on the wall of intrarenal blood vessels through ROS production [105]. Moreover, during conditions of ischemia/reperfusion damage in the kidneys, H_2_O_2_ formation by monoamine oxidase enzymes induced a pro-apoptotic cascade in proximal tubular cells [106]. Finally, SNS may induce inflammation by noradrenaline-mediated cytokine production from liver [107] and heart [108].

In both renal and heart failure conditions, a state of chronic inflammation is present, as documented by elevated levels of C-reactive protein and of several pro-inflammatory cytokines such as IL-1b, IL-6, TNF-a [109–112]. They are able to stimulate renin as well as noradrenaline secretion [113,114]. IL-6 induces upregulation of the Angiotensin type 1 receptor (AT1) and Angiotensin II-mediated ROS production in cultured rat vascular smooth muscle cells, further supporting a link between inflammation, RAAS activation and oxidative stress [115].

Moreover, accumulating evidence suggests that volume overload and venous congestion are an additional source of inflammatory mediators [116,117]. The vascular endothelium itself may be a primary source of cytokine production in response to biomechanical stress due to intravascular congestion [118]. These findings support the potential role of circulating cellular sources of ROS and/or circulating agonists of local sources of ROS in the development of cardiorenal syndrome.

Based on the above reported evidence, oxidative stress appears to play a pivotal role in CRS, since the balance between NO and ROS shifts towards the latter by increased production of ROS and lower availability of NO.

**Figure 3:**
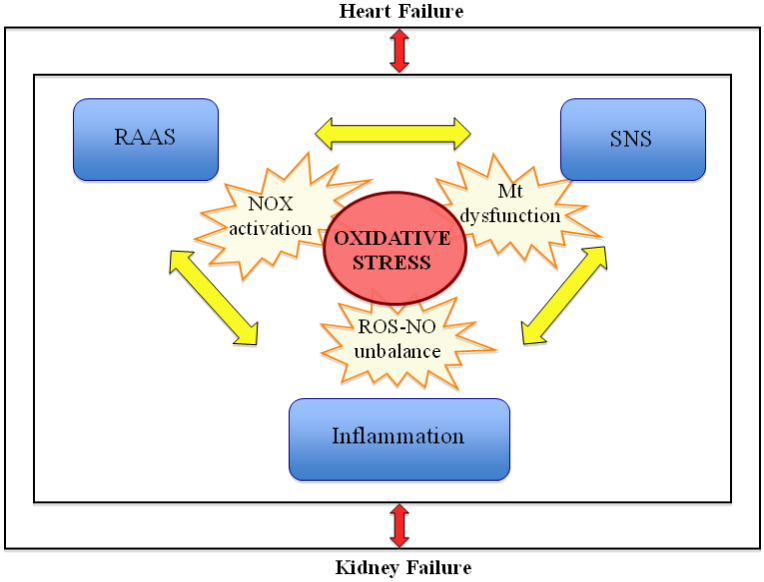
Mechanisms involved in the pathogenesis of chronic cardiorenal syndrome. When either the heart or kidneys fails, a vicious cycle develops: the renin-angiotensin-aldosterone system, the sympathetic nervous system and the inflammatory process interact and synergize to lead to NADPH oxidase activation, mitochondrial dysfunction and NO-ROS unbalance, ultimately causing increased oxidative stress and further impairment of organ function. Legend: NOX = NADPH oxidase; RAAS = renin-angiotensin-aldosterone system; SNS = sympathetic nervous system; ROS = Reactive oxigen species; NO = nitric oxide; Mt = mitochondria.

An imbalance among the RAAS, SNS and inflammation rate may certainly promote and accelerate CRS development (Figure 3).

## 6. Targeting Oxidative Stress to Treat CRS: Anything on the Horizon?

### 6.1. Drugs for HF with an Indirect Effect on Oxidative Stress

Angiotensin-converting enzyme inhibitors (ACEI), angiotensin receptor blockers (ARB), and aldosterone receptor antagonists, as well as β-blockers, have clearly emerged as cornerstones of chronic HF therapy [119–129]. The renoprotective effect of ACEI and ARB has been well documented both in diabetic and in nondiabetic patients [124–126]. On the other hand, β-blockers play a pivotal role in interrupting the sympathetic overflow observed in congestive HF. Their action is at least partly due to improvement of myocardial energetics [130] and to reduction of oxidative stress in the myocardium [131]. Very little is known about their effects on renal function.

### 6.2. Oxidative Stress and NADPH Oxidase: Promising Therapies

ACEI, ARB and statins have been shown to modulate indirectly NADPH oxidase function [13]. Most importantly, promising results may be foreseen either by directly targeting NOXs or by modulating their function [13]. Unfortunately, currently available NOXs inhibitors appear to have low specificity and they are toxic for clinical use [13].

Despite the unfavourable evidence, the search for successful inhibitors continues in order to allow the introduction of new molecules into clinical practice [132]. Strategies acting directly on NADPH oxidases are different: direct inhibition of NADPH oxidase expression; blockade of translocation of its cytosolic subunits to the membrane; inhibition of the p47phox subunit either by preventing its phosphorylation through PKC inhibitors or by blocking its binding to other subunits. Both a decrease of signal transduction and inhibition of Rac 1 translocation have also been shown to decrease ROS generation [133].

Diphenyliodonium, one of the first inhibitors used in model studies, exerts a potent effect but lacks specificity [134]. Later studies investigated apocynin, a naturally NADPH oxidase oral inhibitor that blocks NADPH oxidase assembly [135]. Furthermore, a molecule called S17834 was introduced: it inhibits NADPH oxidase activity in endothelial cells and exhibits potent anti-inflammatory properties including the ability to reduce expression of redox-sensitive genes such as *VCAM-1*. Its major mechanism of action involves prevention of cytosolic (mainly p47phox) binding to the enzyme membrane complex. S17834 has no effect on superoxide produced by xanthine oxidase, indicating that it does not have significant superoxide scavenger properties [136]. Based on recent studies, S17834 decreases apoptotic signaling [137]. An additional selective NADPH oxidase inhibitor is the gp91ds-tat that binds to the p47phox subunit and prevents its interaction with other subunits. Therefore, it is able to inhibit NOX1 and NOX2, but it does not inhibit NOX4, since NOX4 activity is p47phox-independent [138].

In summary, the selective inhibition of NADPH oxidases appears to be a promising approach, with the potential to be far more efficient than non-selective scavenging of ROS through the administration of chemical antioxidants (vitamins).

### 6.3. Oxidative Stress and Mitochondria: Promising Therapies

As stated above, mitochondrial dysfunction may be an important event in the development of both cardiac and renal damage. Thus, mitochondria are taking the center stage in the search for novel cardioprotective therapies, as their dysfunction appears early in the development of both HF and renal damage [139].

In an elegant review, Bayeva *et al*. identified the maintenance of mitochondrial biogenesis against cardiac insult and the reduction of mitochondrial ROS production as the two most promising approaches that may soon yield effective treatments for HF [140].

Currently, no drugs that specifically target mitochondrial biogenesis in HF are available. However, promising results may be foreseen by targeting adenosine monophosphate–activated kinase (AMPK) and endothelial nitric oxide synthase (eNOS).

Strategies acting directly on ROS, by either reducing the production or increasing the elimination, can be an important therapeutic target for the treatment of HF. Several trials evaluated the efficacy of chemical antioxidants in the treatment of HF, but the results were disappointing. Clinical trials such as HOPE [141] and HPS [142] failed to show a benefit of vitamin C and vitamin E administration for the treatment of cardiovascular diseases, including HF.

Interestingly, MitoQ represents a promising molecule for its antioxidant role exerted both in the kidneys and in the heart. MitoQ is a mitochondria-targeted antioxidant, designed to accumulate in mitochondria *in vivo* and to protect from oxidative damage. Ubiquinone is the active antioxidant component of MitoQ. The lipophilic part of MitoQ enables the molecule to accumulate selectively in mitochondria, whereas other antioxidant molecules are distributed through the whole cell. Tracer studies found the compound to be rapidly taken up into the heart, liver, brain, kidneys, and muscles, with the highest accumulation observed in heart and liver [143]. *In vivo* studies have shown that administration of MitoQ to rats for two weeks reduced oxidative stress and protected the heart against ischemic-reperfusion injury. MitoQ preserved cardiac function in a spontaneous rat model of HF [144,145].

Recent studies demonstrated that MitoQ has also a role in protecting the kidneys from oxidative stress damage. In fact, this molecule decreased renal tubular damage and cell death during cold preservation in porcine kidney [146], thus behaving as a promising therapeutic tool in the setting of renal failure and potentially also in that of CRS.

## 7. Conclusions and Outlook

Apart from hemodynamic factors, an imbalance between RAAS, SNS and inflammation underpins the development and progression of CRS through a vicious circle that leads to and amplifies oxidative stress production. Although several interventions were shown to be effective in reducing oxidative stress, at least in experimental studies, no effective strategies to directly influence the known sources of ROS are currently available for widespread clinical use.

A thorough understanding of the cellular and mitochondrial pathways involved in both renal and cardiac oxidative stress is essential in order to allow the development of novel and more effective therapeutic strategies able to improve survival and prognosis in CRS patients. Moreover, clinical trials on the treatment of CRS are strongly needed.
